# Endovascular biopsy in neurointerventional surgery: A systematic review

**DOI:** 10.1177/15910199241240508

**Published:** 2024-03-22

**Authors:** Oleg Shekhtman, Georgios S Sioutas, Gennadii Piavchenko, Shubhang Bhalla, Daniel L Cooke, Ethan Winkler, Jan-Karl Burkhardt, Visish M Srinivasan

**Affiliations:** 1 Department of Neurosurgery, Perelman School of Medicine at the University of Pennsylvania, Philadelphia, PA, USA; 2 Department of Human Anatomy and Histology, Sechenov University, Moscow, Russia; 3 189227 Department of Neurological Surgery, University of California San Francisco, San Francisco, CA, USA; 4 Department of Interventional Radiology, University of California San Francisco, San Francisco, CA, USA

**Keywords:** Endovascular biopsy, intracranial aneurysms, single cell RNA sequencing (scRNA-seq), fluorescence-activated cell sorting, endothelial cells

## Abstract

**Introduction:**

Endothelial cells (ECs) continuously line the cerebrovasculature. Molecular aberrations in the ECs are hallmarks and contributory factors to the development of cerebrovascular diseases, including intracranial aneurysms and arteriovenous malformations (AVMs). Endovascular biopsy has been introduced as a method to harvest ECs and obtain relevant biologic information. We aimed to summarize the literature on endovascular biopsy in neurointerventional surgery.

**Methods:**

We conducted a comprehensive literature search in multiple databases, identifying eligible studies focusing on neurosurgical applications of endovascular biopsy. The systematic review followed the Preferred Reporting Items for Systematic Reviews and Meta-Analyses (PRISMA) statement. The relevant information was collected, including study characteristics, biopsy techniques, and key findings.

**Results:**

Nine studies met the inclusion criteria and were included. The studies involved the collection of ECs using various endovascular devices including coils, guide wires, different stents, and forceps. Endothelial-enrichment techniques, such fluorescence-activated cell sorting (FACS), collected ECs and facilitated downstream applications of bulk or single-cell RNA sequencing (scRNAseq). The studies provided insights into gene expression profiles and identified potential biomarkers associated with intracranial aneurysms. However, challenges were observed in obtaining an adequate number of ECs and identifying consistent biomarkers.

**Conclusion:**

Endovascular biopsy of endothelial cells (ECs) in cerebrovascular pathologies shows promise for gene expression profiling. However, many studies have been limited in sample size and underpowered to identify “signature genes” for aneurysm growth or rupture. Advancements in minimally invasive biopsy methods have potential to facilitate applications of precision medicine in the treatment of cerebrovascular disorders.

## Introduction

Endothelial cells (ECs) form a continuous lining of the lumen of the cerebrovasculature to tightly regulate the cellular and molecular exchanges between the brain and circulation—known as the blood–brain barrier.^1^ Recent studies have highlighted the significant role that ECs play in the maintenance of homeostasis, metabolism, and regeneration. Tissue-specific ECs are crucial in supplying both activating and inhibiting growth factors, extracellular matrix components, as well as regulating vascular tone and controlling immune cell trafficking. Together, these functions are collectively referred to as paracrine or angiocrine regulation.^2–5^

The available evidence suggests that injury to the endothelial cell (EC) layer is the initial step in the development of intracranial aneurysms (IAs). Multiple mechanisms contribute to IA formation, including hemodynamic stress, oxidative stress, NOS dysfunction, estrogen imbalance, and endothelial cell-to-cell junction compromise.^6,7^ The onset of pathological changes in the ECs, inflammation, and aneurysm dilation are strongly related to hemodynamic stress.^8–10^

A technique for harvesting viable vascular endothelium utilizing stainless-steel guide wires for the purpose of gene expression analyses, now known as endovascular biopsy, was first described by Feng in 1999.^11^ The procedure is an addition to diagnostic or interventional procedures, only shortly increasing operation time. It utilizes standard endovascular instruments, such as guide wires, coils, and stents. Recent studies have adapted this technique for the cerebrovasculature and shown that endovascular biopsy allows for collection of several hundred ECs with utilization of cell enrichment strategies, such as fluorescence-activated cell sorting (FACS).^12–16^ Applications of innovative next-generation sequencing technologies, such as bulk RNA sequencing (RNAseq) and single-cell RNA sequencing (scRNAseq), have yielded preliminary insights to molecular aberrancies in multiple cerebrovascular pathologies from living human patients, such as aneurysms or vascular malformations.^12,13,17,18^ In this study, our objective was to conduct a systematic review and summarize the literature on endovascular biopsy within neurosurgery, elucidating its benefits for both clinical practice and research endeavors.

## Materials and methods

### Search design and literature search strategy

This systematic review was conducted according to the Preferred Reporting Items for Systematic Reviews and Meta-Analyses (PRISMA) statement.^19^ Eligible studies were identified through search of the PubMed, Web of Science and Scopus, and Embase databases (end of search date: March 3, 2023). The literature search was conducted by two independent investigators (OS and GS), using the following algorithm: “endovascular biopsy” OR “endovascular catheter biopsy” OR “catheter biopsy” OR “endoluminal biopsy.” Original clinical studies, published in English, and focusing on neurosurgical applications were only deemed eligible. The reference lists of studies included in the systematic review were also hand-searched for missed studies. Exclusion criteria were: (i) articles published in a language other than English, (ii) papers irrelevant to the field of neurosurgery, (iii) narrative reviews and meta-analyses, (iv) letters to the editor, errata, comments, and editorials, and (v) conference abstracts. All disagreements were resolved by consensus with a senior author (VMS).

### Data extraction and tabulation

Standardized, pre-piloted forms were used for data extraction and quality assessment of eligible studies. Data extraction was performed by two independent reviewers (OS and GS) and any discrepancies were identified and resolved through quality control discussions with another author (VMS) whenever necessary. We extracted the following data from the eligible articles: study characteristics (first author, publication year, study material, collection site, biopsy device), additional study techniques, endothelial cell yield, and key findings.

## Results

**Figure 1.**
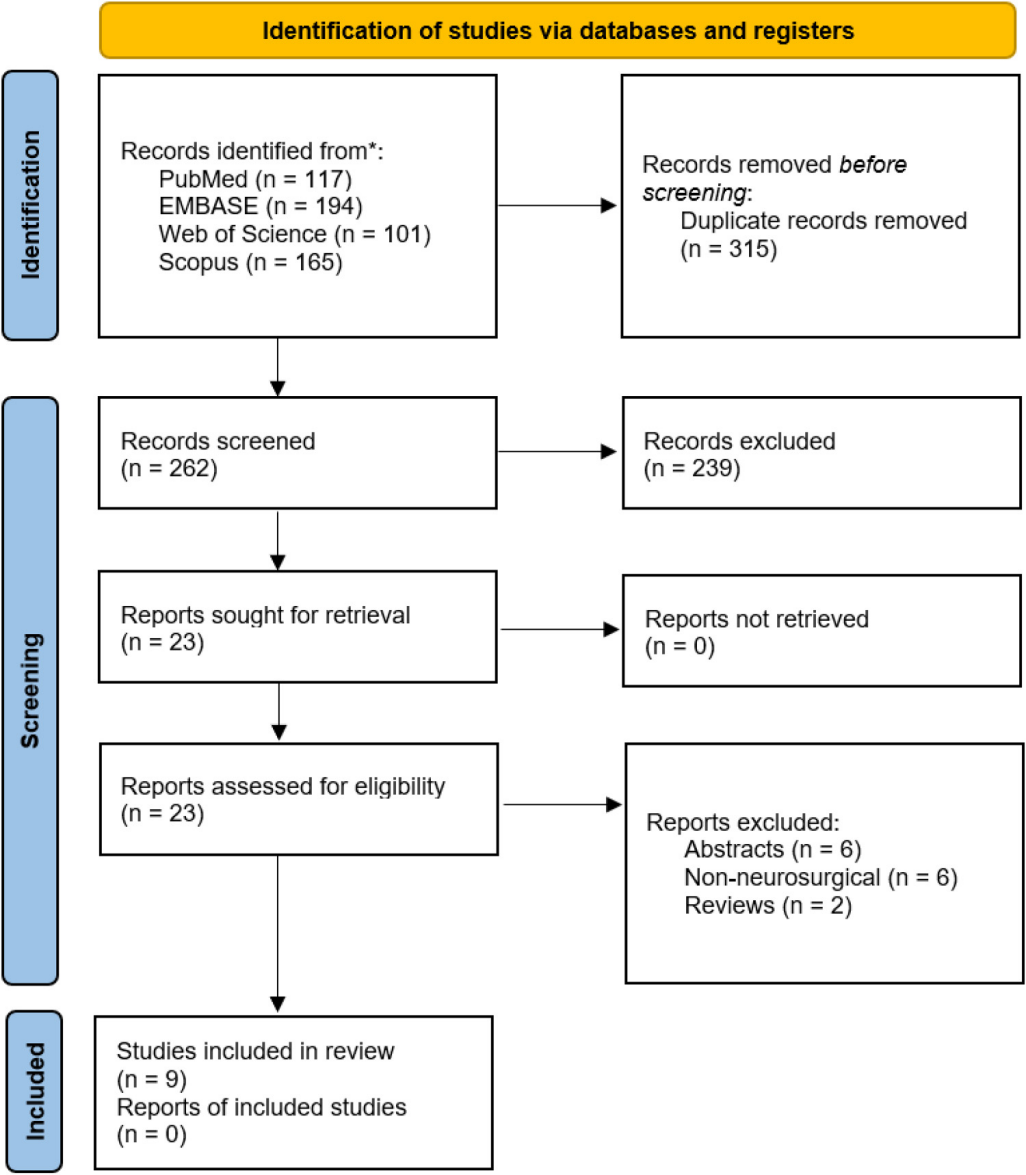
Preferred Reporting Items for Systematic Reviews and Meta-Analyses (PRISMA) 2020 flow diagram for study selection.

The initial literature search yielded 577 potentially relevant articles (end-of-search date: March 3, 2023). After title/abstract screening, 23 articles were retrieved for full-text evaluation. Overall, nine studies^11–16,18,20,21^ fulfilled the pre-determined inclusion criteria and were included in the present systematic review. Study characteristics are summarized (Figure 1). Seven studies were conducted in patients, one study was conducted in animals, and one study included patients and rabbits. From 1999 to 2022, eligible human-based studies enrolled a total of 89 patients, while animal-based studies included 78 rabbits.

The site of EC collection was arteries (iliac, carotid, MCA, etc.) in six studies, sinuses and veins in two, and a combination of both in one. Multiple endovascular devices were used to harvest endothelial cells, including platinum coils, guide wires, different stents, and forceps. A summary of the main experimental conditions and key findings is presented in Table 1.

**Table 1.** Studies included in the systematic literature review.

Author, year	Subjects	Collection site	Biopsy device	Study technique	EC yield	Key findings
Feng, 1999^11^	29 patients with vascular diseases	Iliac and carotid arteries, large veins	Guide catheter	Reverse PCR and ICC	262 cells ± 45 (range, 30–880)	Endothelial sampling is feasible and safe. Expression of VCAM-1 transcripts increased with age
Cooke, 2015^14^	36 rabbits (elastase aneurysm model); coils – 14, stents – 15, controls – 7	Carotid ligation aneurysm (coils), rabbit aorta (stents)	Coils, intracranial stents, and stent-like devices	FACS, PCR	7.93 ± 8.33 coil, 831.33 ± 887.73 – stents	Coil stiffness, stent-to-aorta diameter ratio, stent length, and the use of a pulling retrieval technique increased EC yield
Quan, 2018^20^	9 patients with venous lesions	Sigmoid, transverse sinus, jugular vein	Biopsy forceps device	Cell pathology	–	PEB is a safe and efficient alternative procedure
Sheth, 2018^15^	3 patients with acute ischemic stroke	MCA	Stent retriever	FACS, cell pathology	6.8% (±0.68%) of cellular fraction	Novel, minimally invasive biopsy technique to harvest ECs from stent retrievers during ET
Cooke, 2018^13^	10 patients with aneurysms	Aneurysm sac, iliac artery (control)	Coil	FACS, RT-PCR	5.8 ± 6.2 (CD31, 34, 105 positive)	Based on genetic expression no unique cell clustering for aneurysm was found. Overall, method shows promises
Alexander, 2019^16^	32 patients with various lesions, 38 rabbits	Multiple locations (cerebral, pulmonary, renal, aorta)	Multiple devices (coils, balloons, stents, delivery catheters, microwires, etc.)	FACS	3.8–15	Phenox clot retriever and retrievable stents yielded the most total ECs
Narsinh, 2021^12^	Case report	Fusiform vertebrobasilar aneurysm	Coil	FACS, scRNAseq	24	scRNAseq is a feasible technique to study aneurysm pathogenesis
Winkler, 2022^18^	4 patients with brain AVM	juxtaposed arterial feeder	Coil	FACS, RNAseq, CFD	269.0 ± 79.9	Compared to excised AVM, endoluminal biopsy detected 83.3% of genes; strong correlation in gene expression between feeding arteries and nidus was found
Khatibi, 2022^21^	Case report	Sigmoid sinus	Stent retriever and aspiration catheter	Cell pathology	–	The first report to utilize an aspiration catheter for sinus lesion biopsy

AVMs: arteriovenous malformations; CFD: computational fluid dynamic modeling; EC yield: endothelial cell yield; ECM: extracellular matrix; ET: endovascular thrombectomy; FACS: fluorescence-activated cell sorting; ICC: immunocytochemistry; LCM: laser capture microdissection; MCA: middle cerebral artery; PCR: transcription polymerase chain reaction; PEB: percutaneous endovascular procedure; RT-PCR: reverse transcriptase PCR; scRNAseq: single-cell RNA sequencing; VCAM: vascular cell adhesion molecule.

## Discussion

Understanding endothelial function via endovascular biopsy is useful to unlocking various cerebrovascular pathologies. In this systematic review, we identified studies that focused on IAs, arteriovenous malformations (AVMs), ischemic stroke, and genetic cerebrovascular disorders.

Alterations in endothelial function are critical in the development of cerebrovascular pathologies, including IAs and their complications. Thus, it is important to consider using endothelial cells harvested from the zone of interest as a hallmark of molecular changes in the arterial wall. The existing literature provides guidance to possible biomarkers for studying the altered microenvironment of ECs. ECs in the walls of IAs have increased levels of COX-2, microsomal PGES-1, and prostaglandin E receptor 2 (EP2).^22^ Additionally, the activation of the PGE2-EP2 pathway in ECs due to shear stress exacerbates chronic inflammation through NF-κB.^23^ EC-EC junctions, consisting of tight junctions, adherens junctions, and PECAM-1 (CD31), play a crucial role in maintaining vessel wall integrity and regulating paracellular transport. Increasing evidence indicates that EC–EC junction dysfunction (degeneration) contributes to aneurysm pathogenesis.^24,25^

### Initial studies

In the study conducted by Feng et al. in 2011,^11^ samples of endothelial cells were collected from large arteries (internal iliac, carotid) and veins in 29 patients who underwent endovascular procedures using coaxial curved stainless-steel guide wires. They found that the expression of vascular cell adhesion molecule 1 (VCAM-1) is increased significantly with patient age (*p* < .01), but the intercellular adhesion molecule did not show any significant difference. Being the first study, it established initial feasibility and paved the way for further projects. The authors successfully demonstrated the collection of ECs, which were used for rtPCR to assess both transcript and protein expression. Later, in 2013, Cooke et al.^26^ investigated the feasibility of harvesting ECs using detachable coils within the iliac artery of 3 pigs. They collected cells identified with CD31 and Von Willebrand factor (vWF) antibody staining. A positive correlation between coil diameter and endothelial cell counts was observed.

It soon became evident that endovascular biopsy without cell enrichment yields a low number of endothelial cells (EC), limiting further work-up. In 2014, Sun et al.^27^ compared to possible enrichment technologies, laser capture microdissection (LCM) and fluorescence-activated cell sorting (FACS), in a search for a better technique to yield more ECs. Iliac artery EC samples were collected from 10 patients undergoing routine angiography. FACS generated a higher EC yield: 149 ± 56 versus 51 ± 22 by LCM. Moreover, acceptable quantitative PCR results after FACS enrichment were attainable from a single live EC while LCM required minimum 30 ECs to reach acceptable qPCR results.

In their subsequent paper, Sun et al.^17^ collected ECs from the iliac artery of 4 patients and utilized above mentioned FACS and single-cell qRT-PCR to analyze the expression profile of 48 genes. Unsupervised hierarchical clustering separated the 134 ECs into two distinctive clusters: one expressed 19 genes related to angiogenesis, inflammation, and extracellular matrix remodeling, and the other showed similarities to senescent (aging) ECs, which authors explained by natural EC turnover. In 2018, Cooke et al.^13^ published the results of in vivo biopsy in patients with both ruptured and unruptured IAs with the same methodology. A total of 437 FACS-sorted cells were collected, of which 94 (22%) were from aneurysms, averaging 9.4 cells per aneurysm. Linear mixed models, exploratory multilevel component analysis, and self-organizing maps (SOMs) were used for computational analysis. No significant differences were found in expression of 48 selected genes. The possible explanation for this is the small sample size, limited number, or misrepresented gene selection. An important point by the authors is that the gene expression pattern of ECs in an affected artery segment may not be uniform, but rather limited to a specific fraction of cells.^28,29^ These cells, in turn, may not be included in the biopsied material. Therefore, highly specific, and constant biomarkers are needed to make the procedure effective.

### Choosing the right tool

The next step was to explore which instruments are more beneficial in collecting ECs was made by Cooke et al.^14^ Conventional coils produced an average of 7.93 ± 8.33 endothelial cells per coil, while all conventional stents, stent-like devices, and modified stents yielded a significantly higher number of cells, averaging at 831.33 ± 887.73 cells per device. Alexander et al.^16^ pursued the same goal by examining the differences in endothelial cell counts using various devices and sampling locations. Endothelial cells (ECs) were collected from a combined study group (rabbits and humans) with various pathologies (aneurysms, AVMs, extracranial atherosclerotic lesions, and intracranial plaques), as well as 25 control samples from normal vessels. Phenox clot retriever devices and retrievable stents yielded more cells compared to others defices (angioplasty balloons, stent delivery catheters and coils): lesion retrieval comprised 33.3 (*p* < .001) and 63.0 (*p* < .001), respectively. This was explained by the higher radial force exerted by retrievable stents, while coils, although useful for aneurysm sampling, yield fewer ECs due to their smaller profile and less vessel wall interaction.^16^

### Expansion of applications

In the following years, endovascular biopsy was utilized for more than its “classical” use and was applied to different conditions. Quan et al.^20^ presented a brief clinical series on percutaneous endovascular biopsy for venous sinus lesions in patients with intracranial hypertension. Sheth et al.^15^ reported the use of stent-retriever devices for EC collection in patients with large-vessel acute ischemic stroke. The periprocedural endothelial cell biopsy could potentially influence clinical decision-making, as previous studies in animal models have shown the involvement of endothelial dysfunction in the inflammatory signaling during the early stages of ischemia. Their findings are in line with a later report by Khatibi et al.,^21^ who were the first to utilize an aspiration catheter for endovascular biopsy of a sigmoid sinus lesion. The extracted tissue revealed myeloid sarcoma, confirming leukemia recurrence and guiding subsequent targeted oncologic therapy. In all of these studies, the authors were able to safely conduct biopsies as part of the interventional procedure.

### Applying next-gen sequencing

Winkler et al.^18^ described a novel approach combining endoluminal biopsy with computational fluid dynamics and pathology studies to evaluate the RNAseq data in feeding arteries of brain AVMs. By adapting FACS-gating strategy, this study was able to capture several hundred viable ECs per specimen, and the authors benchmarked sequencing approaches from patient-matched samples obtained from endovascular biopsy and tissues from surgical resection (the gold standard). RNAseq analysis demonstrated that endoluminal biopsy detects the vast majority (83.3%) of the genes identified in surgical tissue. The gene expression profiles from both endoluminal and surgically acquired cells showed a strong correlation at the genetic level, indicating a high degree of concordance between the two techniques. A strong correlation in gene expression between feeding arteries and the nidus in surgically resected AVMs was found. Thus, endoluminal biopsy from accessible feeding arteries may become a reliable solution to study molecular profiles of the AVM nidus without additional procedural risk.

An example of another clinically beneficial application is the case report by Narsinh et al.,^12^ who combined EC biopsy with scRNAseq. The authors identified a subset of seven aneurysm-specific ECs that have distinct gene expression patterns. These ECs show increased expression of genes that regulate leukocyte-endothelial cell adhesion, major histocompatibility complex (MHC) class I, T cell receptor recycling, tumor necrosis factor-alpha (TNF-α) response, and interferon-gamma signaling. Based on genetic findings, the authors proposed that certain patients may benefit from anti-inflammatory and immune-modifying drugs as potential therapies for preventing the formation, growth, and rupture of IAs.

### Planning the aneurysm research

Endovascular catheter-based biopsy has evolved over time, and the choice of the best technique depends on the specific goals and circumstances of the procedure. Most studies in neurosurgery have been focusing on evaluating the risk of aneurysm growth or rupture, limiting the analysis to intra-aneurysmal coil biopsies. Coils, as instruments for EC collection, generally yield a lower number of ECs compared to stents or other devices that have increased mural contact. Depending on the research goals, FACS enrichment could be considered the standard procedure for subsequent steps, which may involve bulk, single-cell RNA sequencing, or ATAC sequencing. Endosaccular coil placement does not induce inflammatory activation (migration of circulating monocytes and macrophages) within the aneurysm dome due to the minimal presence of endothelium.^30^ This makes the study of parent arteries of an aneurysm a promising avenue of future research.

### Future directions

Further research involving larger sample sizes is warranted to address the issue of underpowered studies as observed in the past. The comparison of ECs from different sites, including normal vessels and different pathologies, can help identify specific biomarkers associated with different conditions, including aneurysm growth or rupture. Biopsy-acquired cells could be analyzed using cutaneous molecular profiles instead of genome-wide sequencing techniques to identify molecular aberrations.^31^ Combination of endovascular biopsy with novel imaging techniques or “liquid biopsy” also seems promising.^32^ Additionally, future advances in bioinformatics and gene sequencing technologies may open new opportunities to existing approaches. Another avenue is the development of dedicated instruments (“biopsy stents”) designed for targeted biopsy or exploring EC biopsy beyond the “primary” indication for arterial or venous pathologies.

## Conclusion

Gene expression profiling with endovascular biopsy of ECs in cerebrovascular pathologies is a promising technique to obtain more detailed data beyond morphological characteristics. However, identifying “signature genes” associated with aneurysm growth or possible rupture remains challenging. A minimally invasive approach to biopsy aneurysmal tissue can greatly enhance precision medicine strategies for the treatment of IAs. A wide variety of applications beyond aneurysms remains to be evaluated for many other cerebrovascular diseases.

## Abbreviations

COX-2: cyclooxygenase-2
EC: endothelial cells
FACS: fluorescence-activated cell sorting
IAs: intracranial aneurysms
LCM: laser capture microdissection
NOS: nitric oxide synthase
NF-κB: nuclear factor kappa B
PECAM-1: Platelet and endothelial cell adhesion molecule-1
PGES-1: prostaglandin E synthase-1
PGE2: prostaglandin E2 receptor
RNA: ribonucleic acid
rtPCR: reverse transcription polymerase chain reaction
scRNAseq: single-cell RNA sequencing
VCAM-1: vascular cell adhesion molecule 1
vWF: Von Willebrand factor
